# Objective structured clinical examination in basic thoracic ultrasound: a European study of validity evidence

**DOI:** 10.1186/s12890-022-02285-4

**Published:** 2023-01-13

**Authors:** Pia Iben Pietersen, Rahul Bhatnagar, Freja Andreasen, Lars Konge, Christian Borbjerg Laursen, Najib Rahman, Anders Bo Nielsen

**Affiliations:** 1 Department of Radiology, Odense University Hospital, Odense, Denmark; 2 UNIFY - Research and Innovation Unit of Radiology, Department of Clinical Research, University of Southern Denmark, Kløvervænget 10, Entrance 112, 2nd Floor, 5000 Odense C, Denmark; 3 SimC - Simulation Centre, Odense University Hospital, Odense, Denmark; 4 Respiratory Medicine Department, North Bristol NHS Trust, Southmead Hospital, Bristol, UK; 5 Department of Clinical Research, ODIN - Odense Respiratory Research Unit, University of Southern Denmark, Odense C, Denmark; 6 Academic Respiratory Unit, University of Bristol, Bristol, UK; 7 Department of Respiratory Medicine, Odense University Hospital, Odense, Denmark; 8 CAMES - Copenhagen Academy for Medical Education and Simulation, Copenhagen, Denmark; 9 Oxford Centre for Respiratory Medicine, Oxford NIHR Biomedical Research Centre, Chinese Academy of Medicine Oxford Institute, Oxford, UK

**Keywords:** Medical education, Training, Thoracic ultrasound, Ultrasound diagnostics

## Abstract

**Background:**

Basic thoracic ultrasound is being used more frequently by clinicians in several settings due to its high diagnostic accuracy for many common causes of respiratory failure and dyspnoea. However, ultrasound examinations are operator-dependent, and sufficient competences are needed to obtain high sensitivity and specificity of examinations. Additionally, it is crucial for ultrasound operators to perceive the competence to interpret the images and integrate them into the patient history and other examinations. This study aims to explore and gather validity evidence for an objective structured clinical examination test of basic thoracic ultrasound competences and establish a pass/fail score.

**Methods:**

An expert panel created the test which included two theoretical and five practical stations representing cases with different diagnoses that cause respiratory symptoms and which are possible to establish by basic thoracic ultrasound. Twenty-five participants with different levels of experience in basic thoracic ultrasound completed the test. Data of the test scores were used for item analysis, and exploring validity evidence was done according to Messick’s framework which is recommended. The contrasting groups' standard setting method was used to establish a pass/fail score.

**Results:**

The summarised internal consistency reliability was high with a Cronbach’s alpha of 0.87. The novice group (n = 4) had a mean test score of 42 ± 10.1 points, the intermediate group (n = 8) scored 79.1 ± 8.1 points, and the experienced group (n = 13) 89.0 ± 6.2 points (one-way ANOVA, *p* < 0.001). A pass/fail score of 71 points was thus derived (maximum test score = 105 points).

**Conclusion:**

We developed a test for the assessment of clinical competences in basic thoracic ultrasound with solid validity evidence, and a pass/fail standard with no false positives or false negatives.

**Supplementary Information:**

The online version contains supplementary material available at 10.1186/s12890-022-02285-4.

## Introduction

Basic thoracic ultrasound is a versatile and functional point-of-care ultrasound examination that may be useful in the diagnosis and management of a broad range of respiratory conditions, including pneumothorax, pleural effusion, cardiogenic pulmonary oedema, and pneumonia [1–4]. More comprehensive thoracic ultrasound examination can be performed by respiratory physicians, often needing more time and experience but potentially providing information on, for example, lymph node status and pleural pathologies. Basic thoracic ultrasound can be used in several settings, for both adults and pediatric patients, with a high sensitivity and specificity of around 90% [5, 6]. However, as ultrasound findings are highly dependent on the skills of the operator, these high sensitivities and specificities may not be universal, although to date this has not been proven.

Previously, guidelines and recommendations for education and training in basic thoracic ultrasound have relied on an arbitrary number of ultrasound examinations that the trainee must perform, or programs with a predefined duration of time for training [7, 8]. However, these approaches do not guarantee competence, with competence- and mastery learning-based education likely the only way to ensure and reflect true competence [9]. The principle of mastery learning is that all candidates can learn a skill but, due to different learning paces, variations in training time, supervision, and guidance are required. Therefore, it is of crucial importance to assess competences, using tests, to ensure that the expected level is reached before progressing further with education. As such, it is generally recommended that educational curriculums finish with a summative assessment or certification [10].

In 1990, Miller presented a theory and framework for the assessment of clinical competencies [11], emphasising first obtaining sufficient theoretical knowledge (the *“knows how”*) before developing the practical content of the skill or procedure in a learning environment *(the “shows how”*), and last being able to perform the procedure, interpret the results, and integrate the findings into a clinical setting (the *“does”*). For assessment of theoretical knowledge and practical competence in basic thoracic ultrasound, corresponding to the first layers in Miller’s pyramid, studies have been published presenting tests with solid validity evidence [12, 13]. However, to our knowledge, no papers have been published assessing a test for the higher level in the pyramid, the “*shows how”*, in basic thoracic ultrasound.

It is important to ensure that any test implemented measures what it is supposed to measure, and that the test does not pass an incompetent candidate, which has the potential to threaten patient safety. Counter to this, if a test fails a competent candidate, the candidate will waste further training time which is not required.

In this study, we aimed to develop and gather validity evidence for a basic thoracic ultrasound test in the form of an Objective Structured Clinical Examination (OSCE) and, secondarily, to determine a pass/fail score for the OSCE.

## Methods and material

The study took place at two teaching hospitals with extensive experience in thoracic ultrasound training, in Odense, Denmark and Bristol, United Kingdom, between March and July 2022.

### Validity framework

Exploring validity evidence of the OSCE was conducted in accordance with an internationally recommended framework [14, 15], which gathers validity evidence from five sources;

1. Content validity—ensures that the test content reflects what it is intended to measure
2. Response process—ensures uniformity and control of the response and assessments
3. Internal structure—analysis of the quality of the developed test items and subsequent assessment of test reliability
4. Relationship to other variables—correlates the test scores to external variables, in this case, the level of experience
5. Consequences—Establishment of a pass/fail score and exploring the consequences including false positive and false negative

### Preparation and design of the test (content validity)

The content for a basic thoracic ultrasound curriculum was previously established using a Delphi method by an international consensus panel which included some of the authors [12]. Based on this publication and the recommendations for basic thoracic ultrasound promoted by the European Respiratory Society, the content and set-up were established. Two clinical thoracic ultrasound experts and one expert in medical education and competence assessment created the first iteration of the OSCE including seven stations (five practical, hands-on stations, and two theoretical stations). All OSCE material, including the cases, assessment sheets, and instructions were sent to, corrected, and approved by the ERS ultrasound task force group. All OSCE documentation was prepared in English.

An OSCE design was chosen as this method is recommended for the assessment of clinical competences, allowing the complexity of assessing theory, practical skills, and behaviour through direct observation in a standardized environment [16]; the OSCE is thereby a performance assessment with a focus on what the candidate can do rather than what they know [17].

**Fig. 1:**
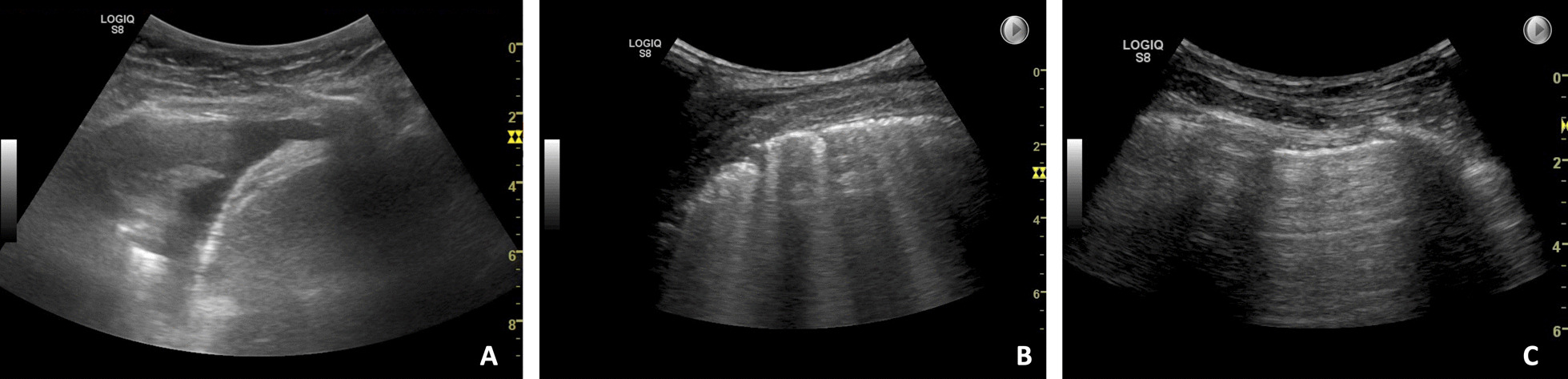
Sonopathological patterns of the pathologies included in the practical stations. **A** Shows a simple, aneccoic pleural effusion, in this case alongside with a minor compression atelectasis of the tip of the lung. **B** Shows multiple B-lines, and when assessed in multiple zones equals interstitial syndrome which can be e.g., cardiogenic pulmonary oedema. **C** Shows the pleurae, and in case of pneumothorax no lung sliding is visible. A lung point will confirm the diagnose of pneumothorax. In the theoretical stations pneumonic consolidations are also a part of the content

**Fig. 2:**
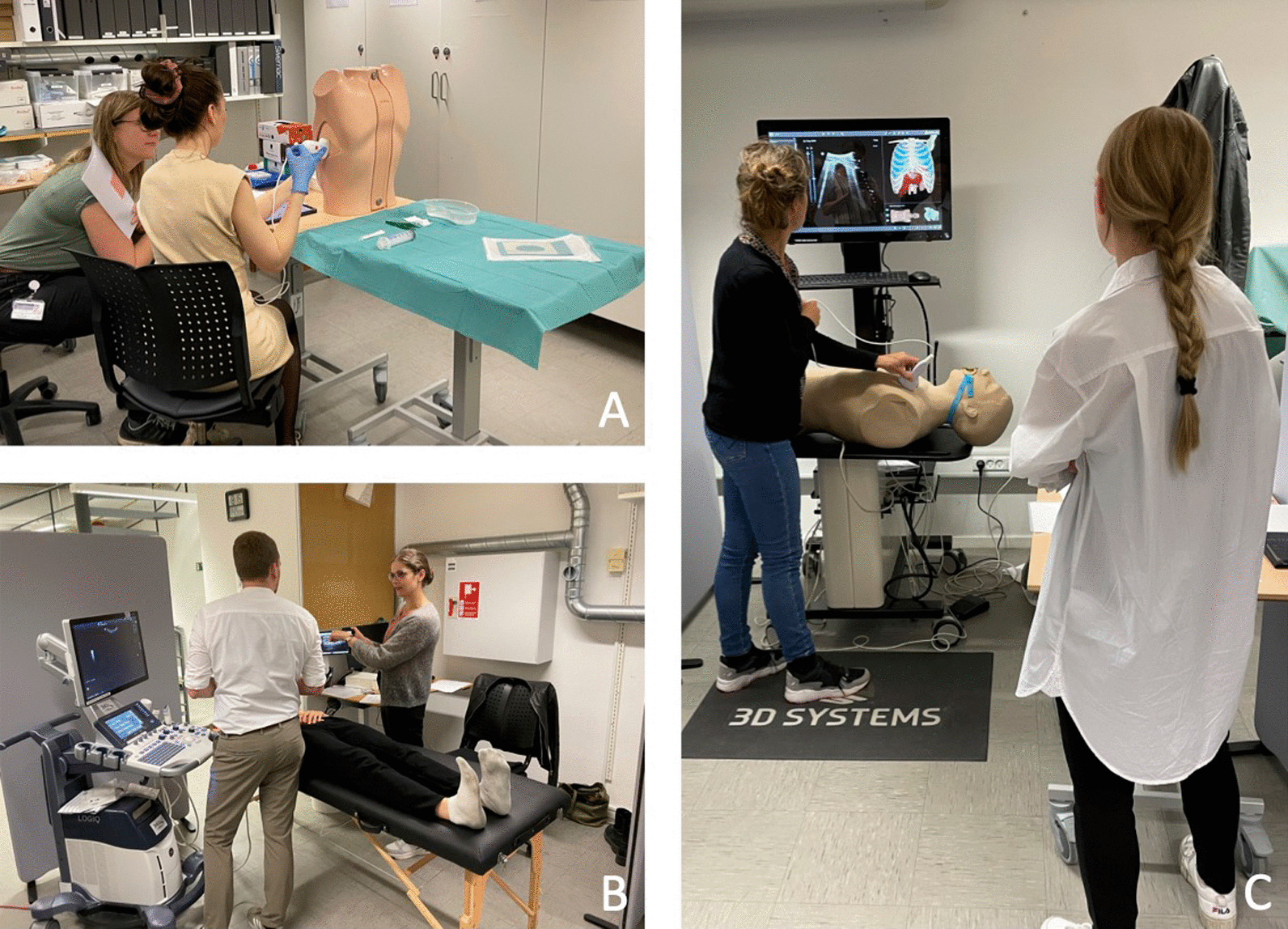
Image **A**: Picture of the pleural aspiration station using a phantom model. The candidate scans the phantom to find the correct position for the aspiration. Image **B**: The candidate (male) has scanned the simulated patient, and the examiner (female) now shows pathological ultrasound clips on a tablet. The candidate must then reflect on the clips and put them into perspective to the patient history and symptoms. Image **C**: The candidate (scanning) scans a simulator which presents with pathological ultrasound images, the examiner observes and asks questions if relevant

Practical stations, involving the use of hands-on skills, included: knobology and image optimization, ultrasound-guided pleural aspiration, and assessments of pleural effusion, interstitial syndrome, and pneumothorax. Figure 1 presents sonopathological patterns of the pathologies included in the stations. Additionally, two theoretical stations were included, each requiring 15 video-based multiple choice questions (MCQ) to be answered [12]. At each practical station, candidates were provided with a short standardised patient history, vital parameters, and a case description. Two stations were simulation-based set-ups, one station using the US Mentor (Surgical Science Simbionix (Gothenburg, Sweden) and one station using the Limb and Things pleural aspiration phantom (Bristol, United Kingdom). The remaining practical stations included actors as simulated patients at which the examiner was able to demonstrate pathological ultrasound clips/images when needed, see Fig. 2. The full equipment list for each station is published as Additional file 2. Station timings were set at 9 min, with one minute for rotation. Over 70 min, it was therefore possible to assess seven candidates per round [18]. No break stations were included.

Assessment sheets were created to include a minimum of 0 points and a maximum of 15 points leaving a total maximum sum score for the test of 105 points.

### Participants

Examiners for the OSCE stations comprised physicians and nurses experienced in the practice and teaching of TUS and pleural intervention. A total of ten examiners were used, each doing two OSCE rounds.

The candidates, whose performance data were used for statistical analyses, were medical students or physicians who were invited using a promotion e-mail to which they responded. The candidates filled out a questionnaire on demographics and experience in basic thoracic ultrasound to categorize them into one of three study groups. The authors predefined the three study groups based on the level of experience with basic thoracic ultrasound: novices, intermediates, and experienced. The *novices* were medical students or physicians with no or limited knowledge of basic thoracic ultrasound and who had performed less than 20 basic thoracic ultrasound examinations. The *intermediates* were medical students or physicians who had theoretical knowledge and clinical experience with basic thoracic ultrasound but who had not performed more than 50 ultrasound examinations on patients in a clinical setting. The *experienced* group was physicians with established knowledge and skills in basic thoracic ultrasound, and who had attended structured courses, have received clinical training in thoracic ultrasound, and had performed more than 50 ultrasound examinations on patients in a clinical setting. The participants and the examiners were blinded to the questionnaires, answers to the questionnaires, and in which study group each participant was categorized into.

### Completion of the test and data collection (response process)

Written instructions for examiners and assessment sheets were prepared for each station. The written instructions for the examiners included direction on what particular basic thoracic ultrasound skill were to be assessed within each station and instructions on how the assessment sheet must be filled out.

Each assessment sheet included seven or eight aspects of the competence assessed (items) which could generate an assessment of 0 points (failed), 1 point (borderline), or 2 points (sufficient) for each item, or in one item 0 points (failed), 0.5 points (borderline), or 1 point (sufficient), see Additional file 1.

The focus was on the instructions being so clear that the assessments could be repeated with high reliability despite changing examiners to minimize assessment bias.

### Statistical analyses

Descriptive statistics are presented as mean and standard deviations, and also range for some results.

The *Internal structure* in Messick’s framework, or internal consistency reliability, measured as Cronbach’s alpha, for all items within each station, was assessed [19]. A high Cronbach’s alpha (determined as 0.70–0.95) shows consistency across the items within each station, and if so, the summarized score for each station would be used for subsequent analyses [20]. A summarized Cronbach’s alpha for all the practical stations was calculated as well to test the reliability across all stations and to ensure a uniform examiner assessment. Based on the total score for each station item analysis was performed.

*Relationship to other variables* was explored by comparing the performance data of the three study groups using a one-way analysis of variance (ANOVA) and between the groups using an independent *t* test.

Evidence for the *consequences* was explored by calculating a pass/fail standard using the contrasting groups’ method on the test score distribution of the novice and experienced competency groups, as well as exploring the number of false-positive, novices ultrasound operators that pass, and false-negative, experienced ultrasound operators that fail [19, 21].

All statistical analyses were calculated using SPSS Version 26 (IBM, Armonk, NY, USA). All statistics were considered significant at a 5% significance level. The specific statistical analyses are described under each topic above.

## Results

Four OSCE sessions were conducted, two in Denmark initially followed by and two in the UK. 28 candidates agreed to attend but, following last-minute cancellations, 25 candidates in total completed the assessments. Four candidates (16%) were novices (all medical students), eight candidates (32%) were categorized as intermediates (one medical students and seven physicians), and 13 candidates (52%) were experienced, thoracic ultrasound operators (one medical student and 12 physicians). The medical student who was categorized as an experienced operator worked in a respiratory department with research in thoracic ultrasound and had been trained for and performed a large number of examinations. The mean age was 32.0 ± 4.8 standard deviation, and most candidates were male (n = 16, 64%).

**Fig. 3:**
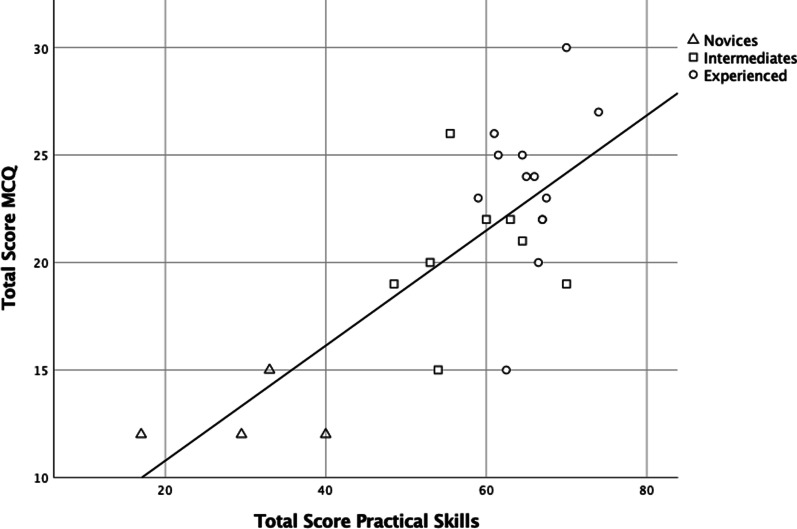
Scatterplot of relationship between practical and theoretical scores. The mean theoretical score of the novices was 12.8, and the mean theoretical score of the intermediates and experienced were 20.5 points and 23.5 points, respectively. The practical mean scores of the novices, intermediates, and experienced were; 29.9 points, 58.6 points, and 65.5 points respectively. Pearson’s correlation coefficient = 0.755, *p* < 0.001

The internal consistency reliabilities for the practical stations are presented in Table 1. The summarized Cronbach’s alpha for all the practical stations was 0.87. Figure 3 presents the relationship between the total practical scores and MCQ scores, divided by study group (novice, intermediate, and experienced).

**Table 1:** Internal consistency reliability results

Station	Station number	Mean score, points	Cronbach’s alpha	Cronbach’s alpha if deleted
1—Knobology	1	11.7	0.84	0.83
2—Pleural effusion	2	10.8	0.84	0.83
3—Interstitial Syndrome	3	11.6	0.85	0.83
5—Pneumothorax	5	10.9	0.88	0.84
6—US-guided pleural aspiration	6	12.6	0.75	0.89
Theoretical stations	4 + 7		0.79	

The table presents the mean scores of each station – independent of the study group, the internal consistency reliability, measured as Cronbach’s alpha, and Cronbach’s alpha if deleted

**Fig. 4:**
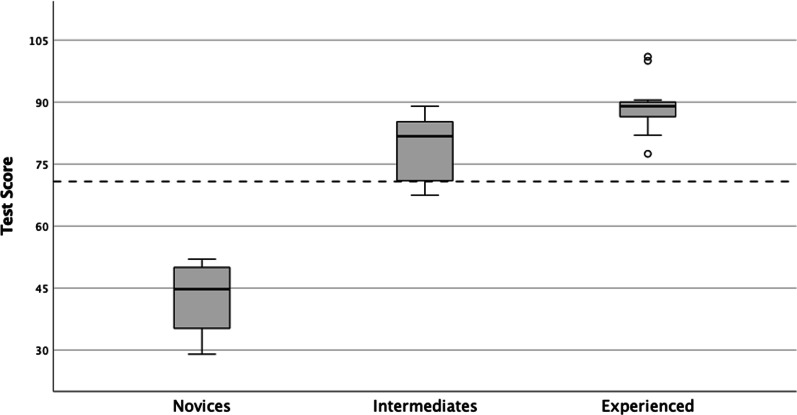
Boxplot of test scores with predefined study groups based on focused thoracic ultrasound experience as the dependent variable and exam test scores as the independent variable. The calculated pass/fail score is presented as the dotted line

The total mean score of the study groups were 42.6 ± 10.1 points for the novice group (range 29–52), 79.1 ± 8.1 points for the intermediate group (range 67.5–89), and 89.0 ± 6.2 points for the experienced group (range 77.5–101). The results are presented as a boxplot in Fig. 4. Comparison using ANOVA showed that the mean scores were significantly different, *p* < 0.001. The independent t-test of the theoretical total score, practical total score, and overall score between the novices and the intermediates, as well as between the novices and experienced were all significant (*p* < 0.001). Comparing mean scores of the intermediate and experienced showed a difference in the theoretical mean score (20.5 points vs. 23.5 points) at 3.0, 95% CI (− 6.3, 0.2) which was not significant, *p* = 0.64, the difference in the practical mean scores (58.5 vs. 65.5 points) was 6.9, 95% CI (− 12.0, − 1.9) which was significantly different, *p* = 0.009, and the difference in overall test score (79.1 vs. 89.0 points) was 9.9, 95% CI (− 16.6, − 3.4) which was also significantly different, *p* = 0.005.

**Fig. 5:**
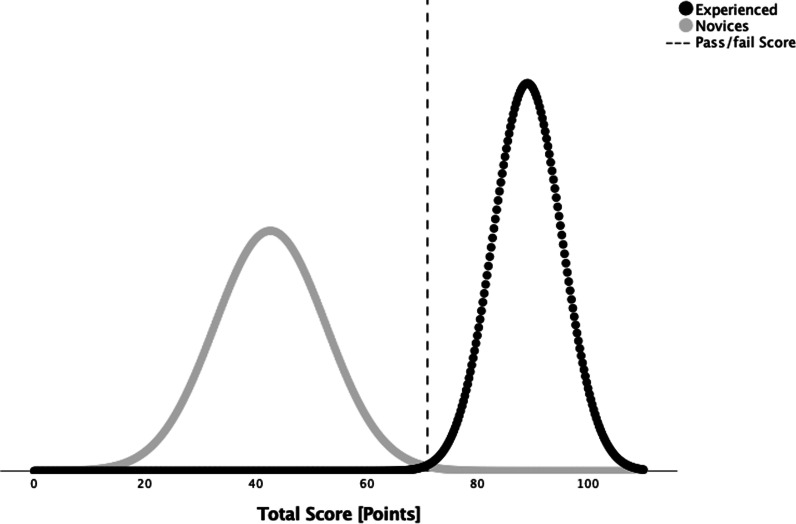
The contrasting group’s method. The normal distribution curves for the mean test score of the novice group (grey curve) and experienced group (black curved) were used to identify the intersection point (dashed vertical line) which is the pass/fail score of 71 points

The normal distribution of the novice and the experienced candidates’ mean scores intersected at 71 points which correspond to the pass/fail according to the contrasting groups’ method, see Fig. 5.

The pass/fail score of 71 points entailed no false positives and no false negatives; no novices passed and no experienced failed the test. In the intermediate group, six candidates passed (75%) and two candidates failed (25%). The results for categorising the study groups in pass or fail were statistically significant using Fisher’s exact test, *p* < 0.001.

## Discussion

We describe the development and validation of an OSCE of basic thoracic ultrasound, which includes assessment of both theoretical and practical competences, as well as the ability to integrate these skills into a clinical context. The test appears able to accurately and reliably distinguish between inexperienced and experienced ultrasound operators.

Due to rapid technological development, basic portable ultrasound machines, and even handheld ultrasound machines, are now widely accessible, making this technique available to a large number of practitioners independent of ultrasound education level or specialty. Structured and clear institutional, national, and international training recommendations and curriculums are required to ensure safe and high-quality practice. These would ideally use both formative and summative assessments, where the formative assessment identifies deficiencies and parts of the competence or skill that need to be trained more, and the summative assessments can be used for certification against a set standard [22].

Competence in basic thoracic ultrasound examination goes beyond just the technical aspects. As mentioned earlier, ultrasound examinations and ultrasound-guided procedures require several different abilities that must be combined to ensure a safe and thorough procedure [23]. Essential aspects are; basic knowledge of thoracic and upper abdominal anatomy, knowledge of ultrasound physics, the ability to technically execute the ultrasound examination, and sufficient clinical aptitude to combine the findings with the patient’s history and symptoms, as well as the other diagnostic tests [24]. Additionally, the ultrasound operator is usually at the bedside during the examination and thus needs communicative competencies and a professional attitude towards the patient and relatives while scanning. Most of these competencies can be assessed using an OSCE, which also allows assessment in a structured and standardized setting [18, 25]. Compared to assessment in a clinical setting, an OSCE using simulated patients ensures the same challenge for each candidate and therefore reduces variability. A disadvantage and challenge of most OSCEs is the complicated set-up required for completing the rounds; including simulated patients and a high number of examiners, with the latter usually requiring experienced clinicians being drawn away from patient care.

It is, however, important to take into account that even though this method assesses the higher layer in Miller’s pyramid, the “*shows how”*, it does not provide a complete profile. Clinical supervision, discussion, and debate always remain relevant even for experienced ultrasound operators, and attaining clinical competence is not a single-point event but a career-long routine. The basic thoracic ultrasound OSCE developed in this study could aid future basic thoracic ultrasound operators’ competencies as part of either formative or summative assessments. The solid validity evidence and results of the study are sufficient and significantly good enough for a summative assessment [19].

There is an increased focus on education, training, and assessment of competences in technical procedures, and thereby an increased amount of published literature on this topic – especially in the post-COVID-19 era [26, 27]. In respiratory medicine, several technical procedures have been the subject of validation studies and research on training-effect [28–30]. The validation studies are often based on phantom- or simulation-based training and assessment, to move the first part of the learning curve into a simulation-based setting instead of a patient-related setting.

Following the work presented here, there is a test of theoretical knowledge, a test of technical skills, and OSCE test of basic thoracic ultrasound competences, all of which could be integrated into a full curriculum. The clinical and patient-related effects of integrating assessments and e.g., simulation-based training and mastery learning approaches, are rarely published. This is due to a huge amount of covariables affecting the clinical and patient-related outcomes, but few studies have been published proving the clinical effect of simulation-based training [31, 32]. In basic thoracic ultrasound this has not yet been proven.

### Strengths and limitations

To our knowledge, this is the first study exploring validity evidence for an OSCE in basic thoracic ultrasound. It is a solid strength of the study that the working and author group are experienced researchers in both thoracic ultrasound and medical education, including assessment, and we rigorously used the methods recommended for validation. Additionally, the author group represents several different specialities that could all benefit from using basic thoracic ultrasound, and thereby this test could easily be implemented in other settings and specialties.

Previously published papers by the group have founded the basis of this study [13, 33, 34], but we acknowledge that the study has several limitations. The number of candidates in the novice and intermediate groups were low compared to what is recommended for validation studies [35]. We only managed to include four novice candidates—all medical students, and eight intermediates (two medical students who have participated in courses and performed basic thoracic ultrasound examinations during clinical training, and six physicians). Despite the low number of candidates, we found significant differences in the mean scores between novices and intermediates and between intermediates and experienced except in the theoretical mean scores. This exception might be due to a statistical type II error (low statistical power) as a previously published validation study of the theoretical test including a higher number of candidates found a significant difference between the intermediates and experienced [12].

Overall, the internal consistency reliability results and Cronbach’s alpha were high, but station 5—US-guided pleural aspiration, had the lowest Cronbach’s alpha of the stations which indicates that the different parts of the performance on this station were not as closely aligned as for the other stations. We believe that this is due to a more heterogenous station that involves both diagnostic ultrasound scanning skills and more technical procedural puncture skills.

We also recognize that it is a limitation that the assessments were done under direct observation which makes the complete blinding of examiners impossible. Additionally, the examiners could be familiar with the level of competence of a specific candidate and could have adjusted their assessment score because they knew that a specific candidate was, for example, a senior physician with experience in basic thoracic ultrasound. Optimally, in validation studies, the candidates should be anonymized and thereby the examiners blinded, but the examiners needed the opportunity to ask questions or make the participants elaborate on specific parts of the examination and decision-making to make an adequate assessment. Hence, totally blinded assessments were not feasible in our setup. Optimally, in validation studies, the candidates should be anonymized and thereby the examiners blinded. This was done in the validation study of chest tube insertion by Hertz et al. (29), however, in a setting like OSCE it would require a heavy set-up with video and the dialogue and possibility for the examiner to ask questions would be missed.

In conclusion, we have developed an objective structured clinical examination in focused lung ultrasound for assessment of competences and gathered multiple sources of solid validity evidence. The results were compact enough for the OSCE to be used as a summative assessment of basic thoracic ultrasound competencies in a mastery learning program due to its discriminative abilities and high reliability.

## Supplementary Information

**Additional file 1**. Assessment sheet items.

**Additional file 2**. Complete list of equipment.

## Data Availability

The datasets used and analysed during the current study are available from the corresponding authors on request.

## Abbreviation

OSCE: Objective Structured Clinical Examination

## References

[1] Dahmarde H, Parooie F, Salarzaei M (2019). Accuracy of ultrasound in diagnosis of pneumothorax: a comparison between neonates and adults-a systematic review and meta-analysis. Can Respir J.

[2] Dubon-Peralta EE, Lorenzo-Villalba N, Garcia-Klepzig JL, Andres E, Mendez-Bailon M (2022). Prognostic value of B lines detected with lung ultrasound in acute heart failure. A systematic review. J Clin Ultrasound.

[3] Chavez MA, Shams N, Ellington LE, Naithani N, Gilman RH, Steinhoff MC (2014). Lung ultrasound for the diagnosis of pneumonia in adults: a systematic review and meta-analysis. Respir Res.

[4] Staub LJ, Biscaro RRM, Kaszubowski E, Maurici R (2018). Chest ultrasonography for the emergency diagnosis of traumatic pneumothorax and haemothorax: a systematic review and meta-analysis. Injury.

[5] Laursen CB, Hanselmann A, Posth S, Mikkelsen S, Videbaek L, Berg H (2016). Prehospital lung ultrasound for the diagnosis of cardiogenic pulmonary oedema: a pilot study. Scand J Trauma Resusc Emerg Med.

[6] Vasquez DG, Berg GM, Srour SG, Ali K (2020). Lung ultrasound for detecting pneumothorax in injured children: preliminary experience at a community-based Level II pediatric trauma center. Pediatr Radiol.

[7] European Federation of Ultrasound in Medicine and Biology. Minimum training requirements for the practice of medical ultrasound in Europe - Appendix 11: Thoracic Ultrasound. 2008. [Available from: https://efsumb.org/wp-content/uploads/2020/12/2009-04-14apx11.pdf.

[8] Royal College of Radiologists. Ultrasound training recommendations for medical and surgical specialties. 3rd ed. 2017. Available from: https://www.rcr.ac.uk/system/files/publication/field_publication_files/bfcr173_ultrasound_training_med_surg.pdf.

[9] McGaghie WC (2015). Mastery learning: it is time for medical education to join the 21st century. Acad Med.

[10] Yudkowsky R, Park YS, Lineberry M, Knox A, Ritter EM (2015). Setting mastery learning standards. Acad Med.

[11] Miller GE (1990). The assessment of clinical skills/competence/performance. Acad Med.

[12] Pietersen PI, Konge L, Madsen KR, Bendixen M, Maskell NA, Rahman N (2019). Development of and gathering validity evidence for a theoretical test in thoracic ultrasound. Respiration.

[13] Pietersen PI, Konge L, Graumann O, Nielsen BU, Laursen CB (2019). Developing and gathering validity evidence for a simulation-based test of competencies in lung ultrasound. Respiration.

[14] Messick S (1987). Validity. ETS Res Rep Ser.

[15] American Educational Research Association APA, National Council on Measurement in Education. Standards for Educational and Psychological Testing. Washington, DC: American Educational Research Association; 2014.

[16] Zayyan M (2011). Objective structured clinical examination: the assessment of choice. Oman Med J.

[17] Harden RM, Gleeson FA (1979). Assessment of clinical competence using an objective structured clinical examination (OSCE). Med Educ.

[18] Harden RM (1988). What is an OSCE?. Med Teach.

[19] Rachel Yudkowsky YSP, Steven M. Downing. Assessment in Health Professions Education 2nd ed. Yudkowsky P, Downing, editor. New York: Routledge; 2020.

[20] Tavakol M, Dennick R (2011). Making sense of Cronbach's alpha. Int J Med Educ.

[21] Jorgensen M, Konge L, Subhi Y (2018). Contrasting groups' standard setting for consequences analysis in validity studies: reporting considerations. Adv Simul (Lond).

[22] Wood DF. Formative assessment. Understanding Medical Education2013. p. 317–28.

[23] Kahr Rasmussen N, Nayahangan LJ, Carlsen J, Ekberg O, Brabrand K, Albrecht-Beste E (2021). Evaluation of competence in ultrasound-guided procedures-a generic assessment tool developed through the Delphi method. Eur Radiol.

[24] Skaarup SH, Laursen CB, Bjerrum AS, Hilberg O (2017). Objective and structured assessment of lung ultrasound competence. A multispecialty Delphi consensus and construct validity study. Ann Am Thorac Soc.

[25] Hodges B (2003). OSCE! Variations on a theme by Harden. Med Educ.

[26] Lee K, Whelan JS, Tannery NH, Kanter SL, Peters AS (2013). 50 years of publication in the field of medical education. Med Teach.

[27] Gordon M, Patricio M, Horne L, Muston A, Alston SR, Pammi M, et al. Developments in medical education in response to the COVID-19 pandemic: A rapid BEME systematic review: BEME Guide No. 63. Med Teach. 2020;42(11):1202–15. 10.1080/0142159X.2020.1807484 32847456

[28] Konge L, Larsen KR, Clementsen P, Arendrup H, von Buchwald C, Ringsted C (2012). Reliable and valid assessment of clinical bronchoscopy performance. Respiration.

[29] Hertz P, Jensen K, Abudaff SN, Strom M, Subhi Y, Lababidi H (2018). Ensuring basic competency in chest tube insertion using a simulated scenario: an international validation study. BMJ Open Respir Res.

[30] Konge L, Clementsen PF, Ringsted C, Minddal V, Larsen KR, Annema JT (2015). Simulator training for endobronchial ultrasound: a randomised controlled trial. Eur Respir J.

[31] Aydin A, Ahmed K, Abe T, Raison N, Van Hemelrijck M, Garmo H (2022). Effect of simulation-based training on surgical proficiency and patient outcomes: a randomised controlled clinical and educational trial. Eur Urol.

[32] Bube SH, Kingo PS, Madsen MG, Vasquez JL, Norus T, Olsen RG (2022). National implementation of simulator training improves transurethral resection of bladder tumours in patients. Eur Urol Open Sci.

[33] Pietersen PI, Laursen CB, Petersen RH, Konge L (2021). Structured and evidence-based training of technical skills in respiratory medicine and thoracic surgery. J Thorac Dis.

[34] Thoracic Ultrasound Monograph. Laursen CB, Rahman NM, Volpicelli G, editors. European Respiratory Society. 2018.

[35] Bloch R, Norman G. Generalizability theory for the perplexed: a practical introduction and guide: AMEE Guide No. 68. Med Teach. 2012;34(11):960–92. 10.3109/0142159X.2012.703791 23140303

