# Supplementary material for: Objective structured clinical examination in basic thoracic ultrasound: a European study of validity evidence
Source: BMC Pulm Med. 2023 Jan 13;23:15. doi: 10.1186/s12890-022-02285-4 (PMC9837899; doi:10.1186/s12890-022-02285-4)
Supplement: Supplementary file 1 — Additional file 1. Assessment sheet items. [file 12890_2022_2285_MOESM1_ESM.docx]

**Assessments sheets**

| **Station 1** |
| --- |
| Adequate explanations to patient prior to and during the examination |
| Able to identify key buttons, functions, and transducers on the US machine |
| *Assessment of superficial structure:*  Optimal choice of transducer |
| *Assessment of superficial structure:*  Optimal choice of preset |
| *Assessment of superficial structure:*  Performs systematic adjustment (primarily, depth and gain) to ensure optimal image quality |
| *Assessment of deep structure:*  Optimal choice of transducer |
| *Assessment of deep structure:*  Optimal choice of preset |
| *Assessment of deep structure:*  Performs systematic adjustment (primarily, depth and gain) to ensure optimal image quality |
| **Overall performance** |
| **Total points** |

| **Station 2** |
| --- |
| Adequate explanation to the patient prior to the procedure |
| Ensures optimal patient positioning for pleural effusion assessment |
| Performs TUS systematically |
| Performs systematic adjustment (primarily, depth and gain) to ensure optimal image quality |
| Correct assessment and demonstration of normal structures and shows understanding of typical placement of pleural effusion |
| Correct assessment of demonstrated ultrasound clips |
| Demonstrates how to identify a site for safe aspiration / drainage of fluid in a large effusion |
| Is able to integrate TUS findings with the patient’s history |
| **Overall performance** |
| **Total points** |

| **Station 3** |
| --- |
| Performs lung ultrasound in line with the focused questions |
| Performs FLUS systematically |
| Can elaborate on the adjustments (primarily, depth and gain) |
| Correct handling of transducer |
| Correct assessment of pleura and shows understanding of lung sliding |
| Correct assessment of B-lines and shows understanding of B-lines and Interstitial Syndrome (IS) |
| Is able to make a diagnosis on the basis of the focused lung ultrasound |
| Is able to integrate FLUS findings with the patient’s history |
| **Overall performance** |
| **Total points** |

| **Station 5** |  |
| --- | --- |
| Adequate explanations to patient prior to and during the examination |  |
| Ensures optimal patient positioning for pneumothorax assessment |  |
| Performs TUS systematically |  |
| Performs systematic adjustment (primarily, depth and gain) to ensure optimal image quality |  |
| Correct assessment and demonstration of normal structures and shows understanding of lung sliding |  |
| Correct assessment of demonstrated ultrasound clips |  |
| Demonstrates how to identify lung point when lung sliding is absent anteriorly on the chest |  |
| Is able to integrate TUS findings with the patient’s history |  |
| **Overall performance** |  |
| **Total points** |  |

| **Station 6** |
| --- |
| Explains appropriate patient positioning |
| Demonstrates correct handling of ultrasound equipment |
| Identifies visible structures correctly (thoracic wall, ribs, lung, diaphragm, fluid) |
| Identifies correct entry point |
| Uses strict aseptic technique |
| Inserts needle over superior border of rip |
| Retrieves 50 ml of pleura fluid |
| Removes the aperture drape |
| **Overall performance** |
| **Total points** |
