# Supplementary material for: Objective structured clinical examination in basic thoracic ultrasound: a European study of validity evidence
Source: BMC Pulm Med. 2023 Jan 13;23:15. doi: 10.1186/s12890-022-02285-4 (PMC9837899; doi:10.1186/s12890-022-02285-4)
Supplement: Supplementary file 2 — Additional file 2. Complete list of equipment. [file 12890_2022_2285_MOESM2_ESM.docx]

**List of equipment**

**Station 1 - Knobology:**

- Ultrasound machine
- Bed
- Gel
- Towels
- Simulated patient

**Station 2 – Pleural effusion:**

- Ultrasound machine
- Bed
- Gel
- Towels
- Simulated patient
- Computer or tablet for presenting pathological ultrasound clips
- Band-aid for marking point of pleural drainage

**Station 3 – Interstitial Syndrome:**

- US Mentor simulator, Lung ultrasound module case 4, (Surgical Science Simbionix (Gothenburg, Sweden)

**Station 4 – Theoretical station part I:**

- Computer for presenting the multiple-choice questions
- Answer sheet part I

**Station 5 - Pneumothorax:**

- Ultrasound machine
- Bed
- Gel
- Towels
- Simulated patient
- Computer or tablet for presenting pathological ultrasound clips

**Station 6 – US-guided pleural drainage:**

- Limb and Things pleural aspiration phantom (Bristol, United Kingdom)
- Ultrasound machine
- Gel
- Gloves
- Draping with hole
- Syringe 20 and 50 mL
- Needle for withdrawing local anaesthetic (18G, reed needle)
- Needle for applying local anaesthetic (21G, green needle)
- Saline
- Saline marked as “local anaesthetic”
- Cotton swaps
- Bandage for applying on the patient following the procedure
- Pigtail catheter – one-step technique (7F)

**Station 7 – theoretical station part II:**

- Computer for presenting the multiple-choice questions
- Answer sheet part II
